# Evidence of redox imbalance in a patient with succinic semialdehyde dehydrogenase deficiency

**DOI:** 10.1016/j.ymgmr.2014.02.005

**Published:** 2014-04-01

**Authors:** Anna-Kaisa Niemi, Candida Brown, Tereza Moore, Gregory M. Enns, Tina M. Cowan

**Affiliations:** a Department of Pediatrics, Division of Medical Genetics, Stanford University, Stanford, CA, USA; b Diablo Valley Child Neurology, CA, USA; c Department of Pathology, Stanford University School of Medicine, Stanford, CA, USA

**Keywords:** SSADH, Oxidative stress, Glutathione, GSH, Mitochondria

## Abstract

The pathophysiology of succinic semialdehyde dehydrogenase (SSADH) deficiency is not completely understood. Oxidative stress, mitochondrial pathology, and low reduced glutathione levels have been demonstrated in mice, but no studies have been reported in humans. We report on a patient with SSADH deficiency in whom we found low levels of blood reduced glutathione (GSH), and elevations of dicarboxylic acids in urine, suggestive of possible redox imbalance and/or mitochondrial dysfunction. Thus, targeting the oxidative stress axis may be a potential therapeutic approach if our findings are confirmed in other patients.

## 1 Introduction

Succinic semialdehyde dehydrogenase (SSADH; EC 1.2.1.24) deficiency is a rare disorder (OMIM 271980) of γ-aminobutyric acid (GABA) degradation caused by mutations in *ALDH5A1*[1], [2]. In this disorder, succinic semialdehyde, the transamination product of GABA, is not converted to succinic acid but instead into 4-hydroxybutyric acid (γ-hydroxybutyric acid, GHB) and other related metabolites including 4,5-dihydroxyhexanoic acids [3]. The diagnosis of SSADH deficiency is based on detecting elevated levels of 4-hydroxybutyric and related metabolites in body fluids including urine [3], [4]. The clinical picture of SSADH deficiency is variable, with the most common manifestations being intellectual disability, expressive language deficits, epilepsy, hypotonia, ataxia, sleep disorders, and psychiatric disturbances [1], [5], [6]. Therapeutic interventions attempted in humans or murine models have included vigabatrin [7], phenobarbital [8], and taurine [9], but no treatment has shown consistent significant efficacy. Thus, novel therapeutic approaches are needed.

The pathophysiology of SSADH deficiency is not completely understood. It is unclear whether elevated GABA, GHB, or a secondary deficiency of tricarboxylic acid (TCA) cycle, or Krebs cycle, intermediates due to lack of conversion of succinic semialdehyde to succinic acid, or some another mechanism contributes to the clinical phenotype of these patients. Mitochondrial dysfunction and oxidative stress have been suggested to play a role by both in vitro and murine model studies [10], [11], [12], [13], [14], [15], [16]. Decreased total radical-trapping potential (TRAP), increased lipid peroxidation and altered antioxidant enzyme activities have been demonstrated in SSADH deficient mice [10], [13], [14], [15], [17]. In addition, patients with SSADH deficiency typically have increased levels of urinary dicarboxylic acids that can sometimes be seen in the presence of mitochondrial dysfunction [2], [3].

Glutathione (GSH) is the most abundant low-molecular-weight thiol, and effectively scavenges free radicals and other reactive oxygen species directly and indirectly through enzymatic reactions. Decreased GSH levels are used as a marker of oxidative stress [18], [19], [20] and have been demonstrated in the liver [14] and in the cerebral cortex of SSADH deficient mice [10], [14], [16]. To date, no such studies have been reported in humans.

To evaluate whether signs of oxidative stress suggested by murine models can be demonstrated in humans, we measured blood glutathione levels longitudinally in a 10-month old patient with SSADH deficiency.

## 2 Patient report and results

The patient was born after an uncomplicated pregnancy to non-consanguineous parents via cesarean section due to prolonged labor. Her birth parameters were normal. At 1.5 months of age, she developed stiffening episodes characterized by appearance of holding her breath, appearing startled, and arm extension. On examination at 1.5 months of age she had hypotonia, head lag, a stiffening episode lasting for seconds, some downward inner movement of the eyes at that point, and an exaggerated Moro reflex and startle response. This exaggerated Moro response was not present anymore at age 4 months. At age 4 months the patient was not able to maintain head control against gravity, but was able to lift her extremities. Deep tendon reflexes were preserved at first evaluation at 1.5 months of age but hyporeflexia was present thereafter.

Brain MRI at age 4 months was normal and has not been repeated. Electroencephalogram (EEG) at age showed waking background composed predominantly of alpha, theta and delta range activity with appropriate anterior to posterior gradient which was lost asleep with the development of high amplitude delta activity with frequent bursts of sharpened alpha/beta range activity highest in amplitude on the fronto/central electrodes. Ophthalmological evaluation was repeatedly normal. Plasma amino acids, plasma total and free carnitine, plasma acylcarnitine profile, creatine kinase, electrolytes, complete blood count, lactate, and thyroid function tests were normal. Urine organic acids showed a marked elevation of 4-hydroxybutyric acid (GHB) as well as abnormal elevations of 4,5-dihydroxyhexanoic acid lactones, glycolic, 3-hydroxypropionic, glutaric, adipic, and 2-hydroxyglutaric acids consistent with the diagnosis of SSADH deficiency. Sequencing of *ALDH5A1* revealed a heterozygous splice site variant c.610-612A>G (IVS3-2A>G) in intron 3, and a heterozygous missense mutation c.1333A>C (p.M445L) in exon 9. Both mutations have been previously reported [8], [21] and are considered pathogenic. Biparental inheritance was confirmed.

The patient was started on vigabatrin (up to 150 mg/kg/d) and taurine (160 mg/kg/d) at 5 months of age. At age 7–8 months she was able to roll over, grab objects, push up to support herself on her arms, hold her head up briefly, and made babbling sounds. On physical examination she continued to have severe diffuse though head lag had improved and an exaggerated startle response was not present.

We measured whole blood GSH levels in our patient by liquid-chromatography tandem mass spectrometry [22]. The GSH level in our patient was 585 μM at age 5 months, 702 μM at age 9 months, and 698 μM at age 10 months (controls: 900 μM ± 140, n = 59). The mean value of all three determinations (662 μM) was significantly different from controls in an unpaired T-test (p = 0.011).

## 3 Discussion

We report a patient with an early-onset SSADH deficiency with low GSH levels in blood suggesting increased oxidative stress. This is consistent with reduced levels of GSH reported in the liver and brain of mice with SSADH deficiency [10], [14], [16]. Our observation further supports the hypothesis of oxidative stress and mitochondrial dysfunction in SSADH deficiency suggested by in vitro studies and experimental models [10], [11], [12], [14], [15], [16]. Administration of GHB, which accumulates in SSADH deficiency, has been demonstrated to induce oxidative stress in the cerebral cortex of rats by increasing lipid peroxidation [16]. Succinic semialdehyde (SSA), the substrate of SSADH enzyme, is a reactive carbonyl and may lead to oxidative stress [13], [23]. Furthermore, SSADH enzyme is also responsible for metabolism of the lipid peroxidation aldehyde 4-hydroxy-2-nonenal (4-HNE), an intermediate known to induce oxidant stress [14], [23]. Decreased activities of mitochondrial complexes I–IV have also been reported in hippocampus of a murine model of SSADH deficiency [16].

Low glutathione levels have been demonstrated in the blood of patients with primary mitochondrial disease [24] as well as in the blood and liver tissue of patients with organic acidemias such as methylmalonic acidemia [24], [25], [26] indicating secondary mitochondrial dysfunction and redox imbalance organic acidemias. Increased lipid peroxidation [14], [15], [23] and low glutathione levels [10], [14], [16] in murine models of SSADH deficiency as well as dicarboxylic aciduria reported in patients [3] and mitochondrial dysfunction in SSADH deficiency, similar to other organic acidemias.

We report on a patient with early-onset SSADH deficiency for which we demonstrate persistently low GSH levels and abnormal elevations of some dicarboxylic acids in urine suggesting possible redox imbalance and/or mitochondrial dysfunction. This is the first such report on human SSADH and consistent with studies from murine models. Targeting the oxidative stress axis, e.g. by supplementing with *N*-acetylcysteine or other antioxidants, may be a viable treatment modality for SSADH deficiency and other organic acidemias if our initial findings are confirmed in other patients.
